# The distribution of a germline methylation marker suggests a regional mechanism of LINE-1 silencing by the piRNA-PIWI system

**DOI:** 10.1186/1471-2156-13-31

**Published:** 2012-04-24

**Authors:** Martin I Sigurdsson, Albert V Smith, Hans T Bjornsson, Jon J Jonsson

**Affiliations:** 1 Department of Biochemistry and Molecular Biology, Faculty of Medicine, University of Iceland, Reykjavík IS-101, Iceland; 2 Department of Genetics and Molecular Medicine, Landspitali-University Hospital, Reykjavik IS-101, Iceland; 3 Icelandic Heart Association, Kopavogur IS-201, Iceland; 4 McKusick-Nathans Institute of Genetic Medicine, Johns Hopkins University School of Medicine, Baltimore, MD 21287, USA; 5 Harriet Lane Pediatric Residency Program, Baltimore, MD, USA

**Keywords:** piRNA, PIWI, LINE-1, Transposable element, Germline, Methylation, Epigenetics

## Abstract

**Background:**

A defense system against transposon activity in the human germline based on PIWI proteins and piRNA has recently been discovered. It represses the activity of LINE-1 elements via DNA methylation by a largely unknown mechanism. Based on the dispersed distribution of clusters of piRNA genes in a strand-specific manner on all human chromosomes, we hypothesized that this system might work preferentially on local and proximal sequences. We tested this hypothesis with a methylation-associated SNP (mSNP) marker which is based on the density of C-T transitions in CpG dinucleotides as a surrogate marker for germline methylation.

**Results:**

We found significantly higher density of mSNPs flanking piRNA clusters in the human genome for flank sizes of 1-16 Mb. A dose-response relationship between number of piRNA genes and mSNP density was found for up to 16 Mb of flanking sequences. The chromosomal density of hypermethylated LINE-1 elements had a significant positive correlation with the chromosomal density of piRNA genes (*r *= 0.41, *P *= 0.05*)*. Genome windows of 1-16 Mb containing piRNA clusters had significantly more hypermethylated LINE-1 elements than windows not containing piRNA clusters. Finally, the minimum distance to the next piRNA cluster was significantly shorter for hypermethylated LINE-1 compared to normally methylated elements (14.4 Mb vs 16.1 Mb).

**Conclusions:**

Our observations support our hypothesis that the piRNA-PIWI system preferentially methylates sequences in close proximity to the piRNA clusters and perhaps physically adjacent sequences on other chromosomes. Furthermore they suggest that this proximity effect extends up to 16 Mb. This could be due to an unknown localization signal, transcription of piRNA genes near the nuclear membrane or the presence of an unknown RNA molecule that spreads across the chromosome and targets the methylation directed by the piRNA-PIWI complex. Our data suggest a region specific molecular mechanism which can be sought experimentally.

## Background

Approximately 70% of CpG dinucleotides are methylated in the human genome [1]. Additionally, non-CpG methylation, primarily of the CWG trinucleotide, has recently been described in the human genome [2]. It accounts for 25% of cytosine methylation in embryonic stem cells, compared to only 0.02% of all cytosine methylation in adult cell lines [2]. DNA methylation has several important functions in mammalian genomes, highlighted by the lethal phenotype of homozygous mutants of DNA methyltransferases [3,4]. These functions include regulation of tissue-specific gene expression [5,6], parent-of-origin expression of imprinted genes [7] and the stable maintenance of the inactive X chromosome in females [8,9].

Transposable elements (TE) comprise 45% of the human genome [10] and their activity threatens genomic stability [11]. The two major active subfamilies of transposable elements are the LINE-1 subfamily of the LINE (long interspersed nucleotide elements) family and the *Alu *subfamily of the SINE (short interspersed nucleotide elements) family [10]. Several defense systems against harmful effects of TE exist, and some of them involve DNA methylation [12]. A hypothesis that DNA methylation is a global defense system against all TE in the human genome [13] has been challenged [14]. Examples of alternative defense mechanisms are families of enzymes interfering with TE activity by altering the properties of the RNA transcripts from TE [12], such as the APOBEC3 family [15]. While some of the APOBEC3 family members can inhibit retrotransposition by both LINE-1 and *Alu *[15], other members are selective for inhibition of only *Alu *[16].

DNA methylation appears to be involved in defense of LINE-1 and long terminal repeats (LTR) in mammals. The transcription of LINE-1 elements *in vitro *is controlled by DNA methylation, at least in vitro [17]. Mice homozygous for mutations in the *DNMT3L *gene loose the methylation of LINE-1 and LTR elements resulting in their increased transcription [18].

A germline TE defense system based on the interaction of piwi-interacting RNA (piRNA) with PIWI (P-element induced wimpy testis) proteins was originally described in Drosophila [19]. Similar protein-piRNA complexes have subsequently been discovered in the mouse and human germline [20], where they seem to mediate defense against mouse IAP (Intracisternal A-particle) and LINE-1 elements via DNA methylation [12]. Male mice with mutated PIWI proteins *(MILI, MIWI2) *are sterile and have increased LINE-1 and IAP transcription [19,21], although female null mutants are fertile [21]. In the germline, PIWI proteins in the cytoplasm cleave transcribed RNAs into piRNA. A ping-pong amplification cycle occurs that increases the relative abundance of TE transcripts in the piRNA pool [12]. The genes coding for the 24-30 nucleotide piRNAs are found in clusters on all chromosomes in both human and mice, with the majority of piRNA genes in each cluster generally arising from the same strand [20]. Their locations in the human and mouse genomes is neither dependent on TE nor gene density [20]. Following the formation of piRNA, a piRNA-PIWI complex moves into the nucleus, where the complex directs *de novo *methylation of LINE-1 and IAP elements [22,23] via an unknown mechanism [19]. piRNA-PIWI appears to have a role in the pathogenesis of human cancer and this is currently the focus of ongoing research [24]. In particular, Hiwi, a member of the piwi gene family, appears to be over-expressed in germline tumors such as seminomas [25]. It is also over-expressed in non-germline tumors such as sarcomas, where high Hiwi expression is associated with a worse prognosis [26]. Increased Hiwi expression in tumors is associated with increased DNA methylation levels, and these tumors respond to inhibitors of DNA methyltransferases [24].

There are at least 208 piRNA clusters in the human genome, on average one cluster every 16 Mb [20]. The distribution of piRNA gene clusters on each human chromosome in the genome suggests that this defense mechanism might be regional with a size range in the range of few tens of megabases. This hypothesis predicts that the piRNA-PIWI defense mechanism preferentially methylates sequences that are in proximity to the piRNA clusters. Here, we present data in support of this hypothesis using our previously published methylated-associatetd SNP (mSNP) surrogate marker of germline methylation [27]. This marker was created by constructing a database of the density of C-T or A-G SNPs within the CpG dinucleotide (mSNPs) from the second generation HapMap dataset of SNPs, under the assumption that the majority of these SNPs are likely to stem from the hypermutability of methylated cytosine in the germline [27].

## Results

### The mSNP density adjacent to piRNA clusters

**Figure 1:**
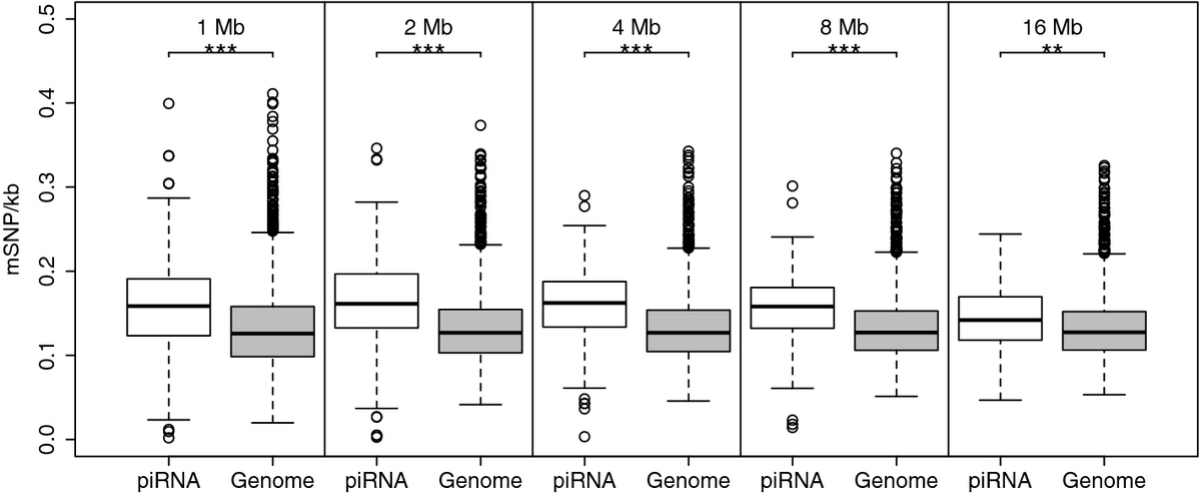
**Box and whisker plots of mSNP density per kilobase in the 1-16 Mb of sequence flanking clusters of piRNA elements (white) compared to the average genome mSNP density (gray)**. Level of significance calculated by Welch's *t*-test are marked with asterisks (*P >*5·10^-2^[NS], *P *< 5·10^-2^[*], *P *< 5·10^-4^[**], *P *< 5·10^-6^[***]). There were significantly more mSNPs flanking piRNA clusters for flank sizes of 1-16 Mb.

If sequences proximal to the piRNA genes are preferentially methylated, we would expect the density of methylation-associated SNPs (mSNPs) to be higher in sequences of flanking piRNA clusters compared to the average genome density. We tested this hypothesis by comparing the mSNP density in 125 kb - 16 Mb of sequences flanking the piRNA clusters with the average mSNP density of the human genome. The mSNP density adjacent to piRNA clusters was significantly higher compared to the genome average for window sizes of 1-16 Mb (Figure 1). In contrast, for 125-500 kb windows, there was no difference in the mSNP density adjacent between piRNA clusters and the genome average (data not shown). There was no notable difference in mSNP density based on strandness of the piRNA cluster (data not shown).

### Dose-response relationship between piRNA elements and mSNPs

**Figure 2:**
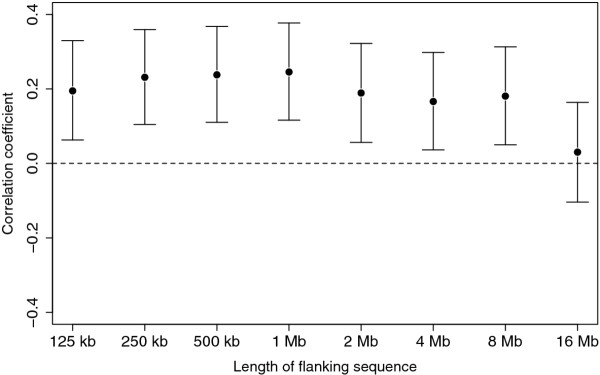
**Correlation coefficient between mSNP density in 125 kb-16 Mb of sequence flanking clusters of piRNA elements and the number of piRNA elements within each cluster**. There was a significant positive correlation between the two variables for flank sizes of 125-8 Mb. For each flank size the point estimate of correlation coefficient is shown and the 95% confidence interval estimated by 10,000 bootstrap estimates of the correlation coefficient.

A hypothesis of a relationship between piRNA genes and mSNP density would be strengthened by demonstrating a dose-response relationship, i.e. that the more piRNA genes per piRNA cluster, the higher density of mSNPs flanking the piRNA cluster. There was a significant positive correlation between number of piRNA genes and density of mSNPs flanking the piRNA clusters for 125 kb - 8 Mb flank sizes (Spearman's ρ = 0.18-0.25, *P *< 0.05 for all sizes). The correlation was stronger with increased flank sizes up to 1 Mb flanks, but then decreased and was lost at 16 Mb flank size (Figure 2).

### Chromosomal distribution of normally methylated LINE-1 elements and hypermethylated LINE-1 elements in the human genome

LINE-1 elements preferentially reside within gene-poor and AT rich regions of the genome [10]. For each LINE-1 element we measured the density of mSNPs in 10 kb flanks on each side of the element. We defined hypermethylated LINE-1 elements as elements with mSNP flank density in the top 10th percentile. The hypermethylated LINE-1 elements had a mean of 8.9 mSNPs in the 10 kb of sequence flanking the elements (median 8, range 7-41 mSNPs). In contrast, the normally methylated LINE-1 elements had a mean of 2.4 mSNPs in the 10 kb of sequence flanking the elements (median 2, range 0-6 mSNPs).

We hypothesized that we might observe a positive correlation between the number of hypermethylated LINE-1 elements and the number of piRNA genes on a chromosomal level. For each chromosome, we calculated the chromosomal density of hypermethylated LINE-1 elements by dividing the absolute number of hypermethylated LINE-1 elements with the chromosomal length. We found that hypermethylated LINE-1 elements were over-represented on chromosomes 6, 10, 13, 17, 18, 20, 21 and 22 and an under-represented on chromosomes 1, 2, 3, 4, 5, 7, 14 and 15 (data not shown). Similarly, we calculated the chromosomal density of piRNA genes by dividing the absolute number of piRNA genes per chromosome with the chromosomal length. There was an over-representation of piRNA genes for chromosomes 6, 10, 17, 19 and 22 and an under-representation of piRNA genes for chromosomes 1, 2, 3, 4, 5, 7, 8, 12, 13, 16, 20 and 21. There was a positive correlation between the chromosomal piRNA gene density and the hypermethylated LINE-1 element density (Spearman's ρ = 0.42, p = 0.05).

### Intra-chromosomal distribution of hypermethylated LINE-1 elements in the human genome and relationship with piRNA clusters

**Figure 3:**
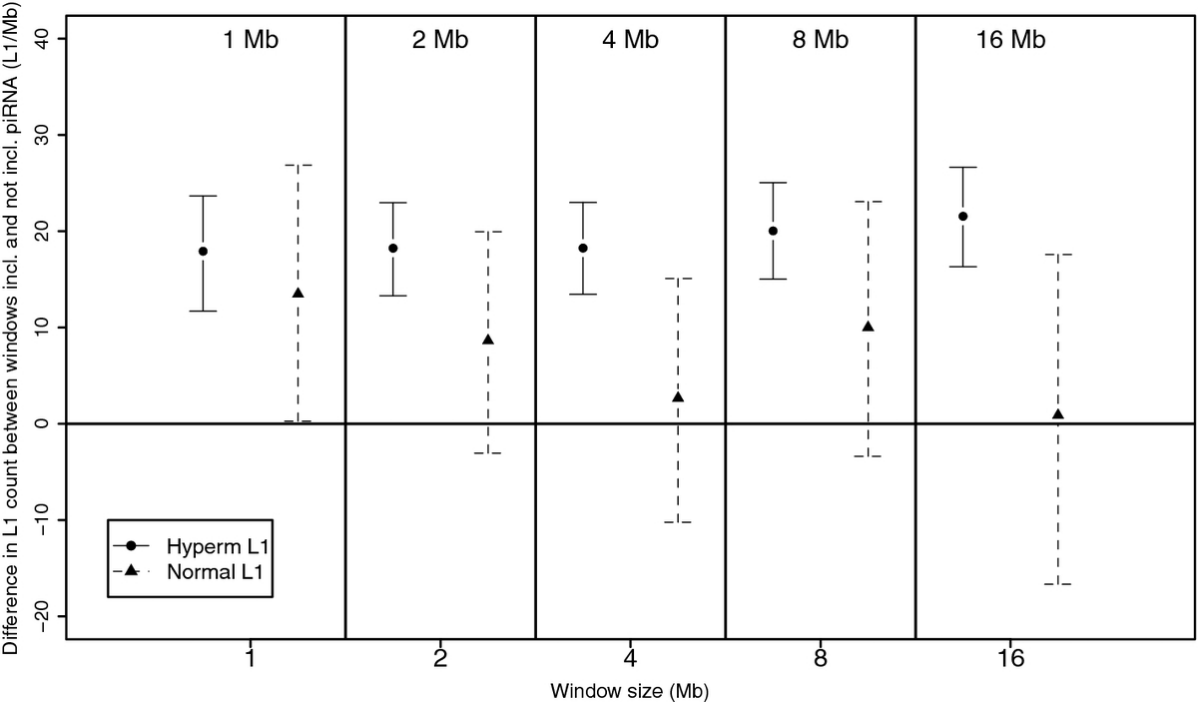
**Difference in LINE-1 counts between genome windows including piRNA clusters and windows not including piRNA clusters for genome windows of 0.5-16 Mb**. Shown is the absolute difference in mean LINE-1 elements per window both for hypermethylated LINE-1 elements (circles, whole lines) and normally methylated LINE-1 elements (triangles, broken lines). Bars represent 95% confidence intervals evaluated by calculating a bootstrap estimates of the differences in means 10,000 times. There were significantly more hypermethylated LINE-1 elements in windows containing piRNA clusters compared to windows not containing piRNA clusters for window sizes of 1-16 Mb. There was no difference in the number of normally methylated LINE-1 elements in windows containing piRNA clusters and windows not containing piRNA clusters for the same window sizes (as the 95% confidence intervals include zero).

Methylation mediated by the PIWI system primarily affects LINE-1 elements. If methylation is increased within sequences adjacent to the clusters, there should be more hypermethylated LINE-1 elements adjacent to piRNA clusters. Figure 3 compares differences in hypermethylated and normally methylated LINE-1 counts between windows including piRNA genes and those not including piRNA genes. For 1-16 Mb windows, there were significantly more hypermethylated LINE-1 in windows containing piRNA genes compared to windows not containing piRNA elements. In contrast, there was no significant difference in counts of normally methylated LINE-1 elements between windows containing piRNA genes compared to windows not containing piRNA genes for window sizes of 1-16 Mb. There was no difference in hypermethylated LINE-1 counts between windows including and not including piRNA genes for window sizes of 125-500 kb (data not shown).

### Distance between LINE-1 elements and piRNA clusters in the human genome

**Figure 4:**
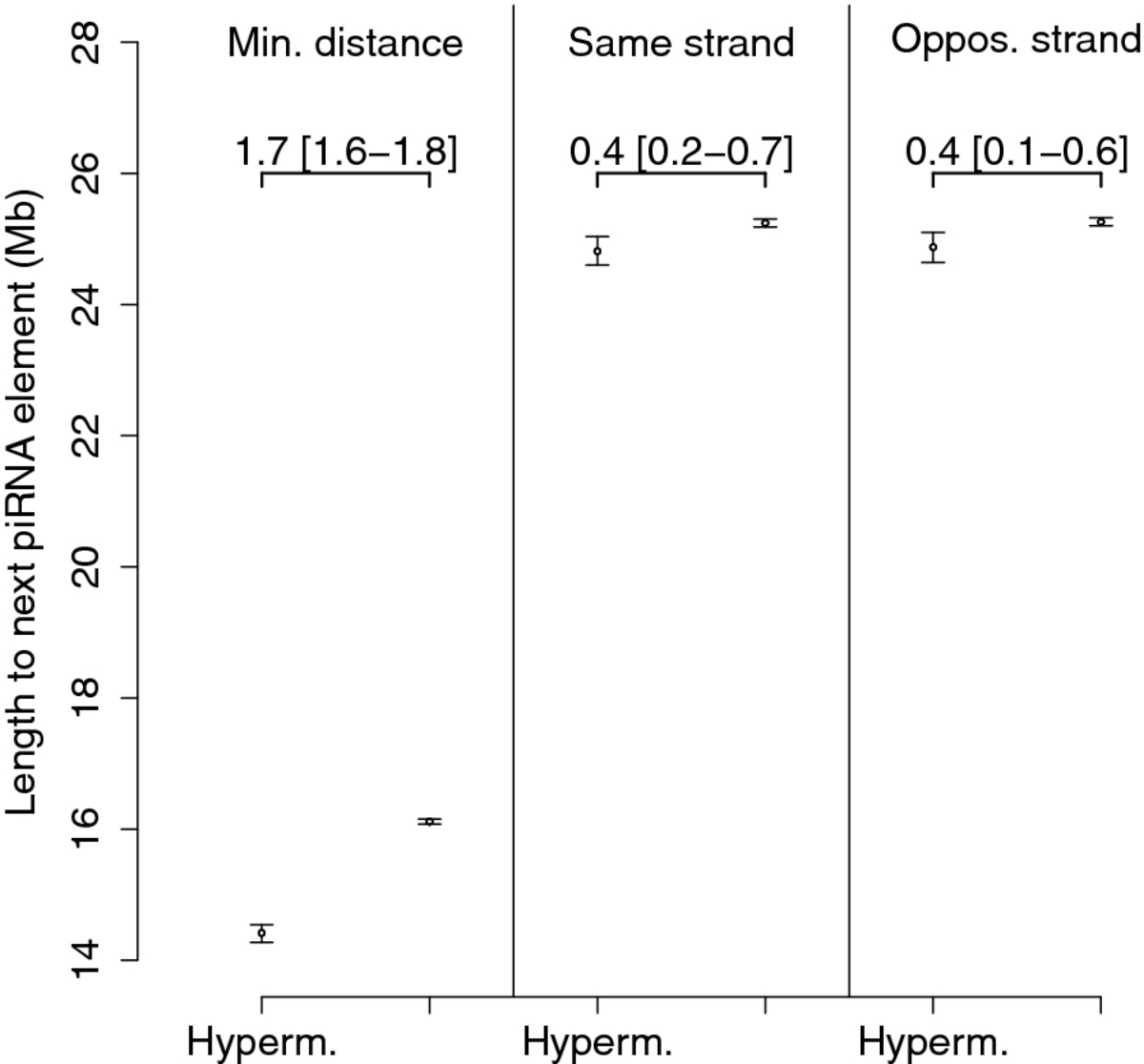
**Mean distances from LINE-1 elements to the next piRNA cluster on the same chromosome for hyperemethylated LINE-1 elements (Hyperm) and normally methylated LINE-1 elements (Normal)**. Shown is the absolute minimum distance (Min.distance) and the mean distance to the nearest piRNA cluster on the same and opposite strand. Bars indicate 95% confidence intervals estimated by calculating a bootstrap estimate of the means 1,000 times. The difference in means and 95% bootstrap interval is shown in the figure. The mean distance to the next piRNA cluster was significantly lower for hypermethylated LINE-1 elements than normally methylated LINE-1 elements, both the absolute minimum distance (14.4 [14.3-14.4] Mb vs. 16.1 [16.1-16.2] Mb), the distance to the nearest piRNA cluster on the same strand (24.8 [24.6-25.0] Mb vs. 25.2 [25.2-25.3] Mb) and the distance to the nearest piRNA cluster on the opposite strand (24.9 [24.6-25.1] vs. 25.2 [25.2-25.3] Mb).

Finally, we hypothesized that if methylation mediated by the PIWI system primarily affects LINE-1 sequences placed proximal to the piRNA clusters, than the distance between the piRNA cluster and the next hypermethylated LINE-1 element should be shorter than the corresponding distance for normally methylated LINE-1 elements. We calculated the distance from each LINE-1 element to the next piRNA cluster on the same chromosome, both the absolute minimum distance and the distance to the nearest piRNA cluster on the same and opposite strand. The absolute minimum distance to the next piRNA cluster was significantly shorter for hypermethylated LINE-1 elements compared to normally methylated LINE-1 elements (Figure 4). Similarly the distance to the nearest piRNA cluster on the same strand an opposite strand was significantly shorter for hypermethylated LINE-1 elements compared to normally methylated LINE-1 elements (Figure 4). There was no difference between the distance to the nearest piRNA cluster on the opposite strand compared to the same strand.

## Discussion

The presence of several piRNA clusters on each mouse and human chromosome [20] might suggest that a great amount of piRNA is needed for the piRNA-PIWI defense system or that the system operates preferentially on sequences proximal to the piRNA clusters via an unknown mechanism. We tested the hypothesis that the piRNA-PIWI system operates preferentially on sequences adjacent to piRNA clusters. Based on the number of the piRNA clusters, the size range of the effect could be within tens of megabases. If this hypothesis is true we should observe more germline methylation of sequences proximal to piRNA clusters. In particular this methylation should be within LINE-1 sequences, the dominant target of the piRNA-PIWI defense system. This hypothesis is testable using the mSNP surrogate marker of germline methylation in the human genome that we have recently described [27]. Our methods only allow us to test this on proximal sequences on the same chromosome, as the spatial organization of the chromosome segments in the human germline is unclear.

We found that the density of mSNPs flanking piRNA clusters was significantly higher for flank sizes of 1-16 Mb compared to the genome average mSNP density. We also found a dose-response relationship between numbers of piRNA genes within piRNA clusters and adjacent mSNPs extending up to 16 Mb. Combined, these results support a hypothesis that there might be an overall more germline methylation adjacent to piRNA clusters, and that the range of the effect is within 16 Mb. The sequences immediately flanking the piRNA clusters (< 1 Mb) might be spared methylation. This could be due to a hypomethylation of the piRNA clusters themselves to maintain their high expression in the germline. Alternatively, our approach might be underpowered to detect a difference in the smaller window sizes.

Second we defined hypermethylated LINE-1 elements based on our mSNP marker and analyzed their distribution in the human genome. We found that the chromosomal density of hypermethylated LINE-1 elements correlated positively with the density of piRNA genes. We also found significantly more hypermethylated LINE-1 elements in genome windows containing piRNA clusters for window sizes of 1-16 Mb. The mean minimum distance to the nearest LINE-1 element was 14-16 Mb, and was significantly shorter for hypermethylated LINE-1 elements. This supports that a higher number of hypermethylated LINE-1 elements is adjacent to piRNA clusters, consistent with a hypothesis that the piRNA-PIWI system might mediate methylation of sequences proximal to the piRNA clusters.

There are several limitations to our approach. The resolution of our mSNP data set limits the sequence length to be analyzed to a minimum of 10 kb. If possible analyzing shorter sequences might be more appropriate, given earlier observations of the methylation spread from retroviruses extending up to 1 kb [28]. We might therefore an expect even a stronger effect if we could limit our selection of hypermethylated LINE-1 elements using methylation information from a shorter sequences.

Our mSNP marker correlates with several other features, including GC ratio, CpG density, repeats density and the absolute density of SNPs [27]. However, many of the observations presented are independent of these effects, such as the dose-response relationship and the chromosomal distribution of piRNA elements and hypermethylated LINE-1 elements. Furthermore the piRNA clusters are neither dependent on gene or repeat density [20].

A potential piRNA-PIWI defense system working on proximal sequences is intriguing, especially given that the amplification cycle and the formation of the piRNA-PIWI complex occurs in the cytoplasm. Perhaps a unique localization signal is transcribed and involved in each piRNA complex? There could also be an RNA molecule that spreads across the chromosomes in both directions from the piRNA clusters and recruits the piRNA-PIWI complex to the region. This could operate in a similar manner to the XIST mechanism of silencing of the X chromosome [29]. Alternatively, actively transcribed piRNA genes might be located near the nuclear membrane, the formation of the piRNA-PIWI complex would occur immediately outside the nucleus and the complex then return to sequences that are most adjacent to the cluster. Our data might also reflect some structural characteristics of chromosomes resulting in correlation between germline methylation, LINE-1 elements and piRNA clusters.

## Conclusions

In summary, our results support an intriguing regional effect of piRNA-PIWI mediated methylation of LINE-1 sequences. These results call for experiments to determine causation and molecular mechanisms.

## Methods

We have previously described the mSNP data set and demonstrated its usage as a surrogate marker for germline methylation [27]. It involves screening large databases of SNPs for mutations likely to stem from mutations of methylated cytosine into thymine (C → T or G → A within a CpG dinucleotide). We created a genome-wide marker of germline methylation from the entire HapMap phase II data set [30] and a dataset of ancestral alleles for the HapMap SNPs [31]. From these data sets, we defined mSNP as any C/T or G/A SNP with adjacent 3' guanine base (for C/T SNPs) or 5' cytosine base (for G/A SNPs). Additionally, we required that the ancestral allele was either cytosine or guanine, excluding T → C and A → G mutations.

The mSNP dataset was validated by demonstrating that it reflected the hypermutability of methylated cytosines and by showing a negative correlation of the marker with CpG islands, sequences generally hypomethylated in the human genome.

For this project, we downloaded the entire human genome sequence from the UCSC data browser http://genome.ucsc.edu[32]. All data was in the NCBI35 (UCSC hg 17) coordinates. This was used to calculate GC ratio and count CpG's. Data on LINE-1 repeats was extracted from the Rmsk table [33] downloaded from the UCSC table browser [34]. The location of piRNA clusters in the human genome was from Girard et al. [20]. Data processing scripts were written in the JAVA programming language, and thoroughly tested prior to usage. All scripts are available at http://www.hi.is/~mis.

All statistics and figure preparation was done in R, version 2.11.0 (The R foundation, Austria). Non-parametric methods were used when the data did not fit normal distribution. Correlation was evaluated by Spearman's ranked correlation. Estimates of means and means differences were assigned 95% confidence intervals by calculating a bootstrap estimate of the parameters and their distribution with 1,000-10,000 random samplings of the data. This was done with the boot package in R. Statistical and figure processing scripts are available upon demand. The level of statistical significance was set at 0.05.

## Competing interests

The authors declare that they have no competing interests.

## Authors' contributions

MIS participated in the design of the study, analyzed the data, drafted the original manuscript and performed critical revision of the manuscript. AVS, HTB and JJJ all contributed to the analysis and interpretation of the data and performed critical revision of the manuscript. All authors have read and approved the final manuscript.
